# The Protective Effects of n-Butylidenephthalide on Retinal Ganglion Cells during Ischemic Injury

**DOI:** 10.3390/ijms23042095

**Published:** 2022-02-14

**Authors:** Yu-Yau Chou, Jia-Ying Chien, Jhih-Wei Ciou, Shun-Ping Huang

**Affiliations:** 1 Department of Molecular Biology and Human Genetics, Tzu Chi University, Hualien 970, Taiwan; 104712123@gms.tcu.edu.tw (Y.-Y.C.); 107712111@gms.tcu.edu.tw (J.-W.C.); 2 Institute of Medical Sciences, Tzu Chi University, Hualien 970, Taiwan; 100712016@gms.tcu.edu.tw; 3 Department of Ophthalmology, Taichung Tzu Chi Hospital, Taichung 472, Taiwan

**Keywords:** ischemic optic neuropathy, retinal ganglion cell, apoptosis, n-butylidenephthalide, NF-kB, neuroinflammation, oxidative stress

## Abstract

Clinically, acute ischemic symptoms in the eyes are one of the main causes of vision loss, with the associated inflammatory response and oxidative stress being the key factors that cause injury. Nonarteritic anterior ischemic optic neuropathy (NAION) is the most common type of ischemic optic neuropathy (ION); however, there are still no effective or safe treatment options to date. In this study, we investigated the neuroprotective effects of n-butylidenephthalide (BP) treatment in an experimental NAION rodent model (rAION). BP (10 mg/kg) or PBS (control group) were administered on seven consecutive days in the rAION model. Rats were evaluated for visual function by flash visual evoked potentials (FVEPs) at 4 weeks after NAION induction. The retina and optic nerve were removed for histological examination after the rats were euthanized. The molecular machinery of BP treatment in the rAION model was analyzed using Western blotting. We discovered that BP effectively improves retinal ganglion cell survival rates by preventing apoptotic processes after AION induction and reducing the inflammatory response through which blood-borne macrophages infiltrate the optic nerve. In addition, BP significantly preserved the integrity of the myelin sheath in the rAION model, demonstrating that BP can prevent the development of demyelination. Our immunoblotting results revealed the molecular mechanism through which BP mitigates the neuroinflammatory response through inhibition of the NF-κB signaling pathway. Taken together, these results demonstrate that BP can be used as an exceptional neuroprotective agent for ischemic injury.

## 1. Introduction

Ischemic optic neuropathy (ION) is one of the main pathological manifestations of visual impairment and vision loss in ophthalmic diseases [1]; among these diseases, nonarteritic anterior ischemic optic neuropathy (NAION) is the most common type [1,2]. The majority of NAION patients are middle-aged and elderly individuals over 50 years of age, who usually have an accompanying risk of cardiovascular disease (hypertension, hypercholesterolemia, nocturnal hypotension, and/or diabetes) [3,4]. Clinically, the most common presentation of NAION in patients includes optic disc swelling and hyperemia, loss of color vision, and visual field defects [2,5], eventually leading to loss of vision. To date, there are still no effective and safe treatment options. However, the NAION model of rodents (rAION) has already been developed for the examination of various potential therapies [6,7,8]. Thus, identifying suitable treatments is an urgent task in preclinical research involving the rAION model. Studies have suggested that circulatory insufficiency in the optic nerve (ON) head leads to the loss of vascular homeostasis, triggering the NAION process. At the same time, disc swelling and compartment syndrome further induce oxidative stress [4,9,10,11,12]. Oxidative stress promotes the development of neuroinflammation and the loss of RGCs [12,13,14,15]. However, the complete mechanism constituting the cause of this chain reaction is still unknown. Thus, reducing the response to neuroinflammation and preventing the loss of RGCs is a priority for preserving visual function.

Angelica has long been used in Chinese medicine to treat arthritis and headaches and, as an antipyretic extract, has been widely used to research pharmacology [16]. n-Butylidenephthalide (BP) is one of the major components of Angelica and has been studied in many different capacities, including for its antitumor [17,18,19], anti-inflammatory [20,21,22], and neuroprotective effects [20,23,24]. In combination with other treatments, BP reduces damage and promotes neurogenesis after cerebral ischemic stroke [25,26]. A study of cardiovascular disorders revealed that BP can regulate the inflammatory process by changing the phenotype of macrophages and preventing myocardial fibrosis in rats after infarction [27]. In terms of neurodegenerative diseases, BP prolongs the life of a mouse model of amyotrophic lateral sclerosis (ALS) by inhibiting motor neurons from apoptosis and reducing the development of neuroinflammation [20]. In summary, BP is a potential candidate for treating ischemic injury. In this study, by modulating inflammatory mediators in ischemic optic neuropathy, we investigated the molecular mechanism of BP. Simultaneously, the survival rates of RGCs and the recovery of visual function were evaluated to confirm the therapeutic effects of BP.

## 2. Results

### 2.1. BP Rescued RGC Survival Rates

**Figure 1:**
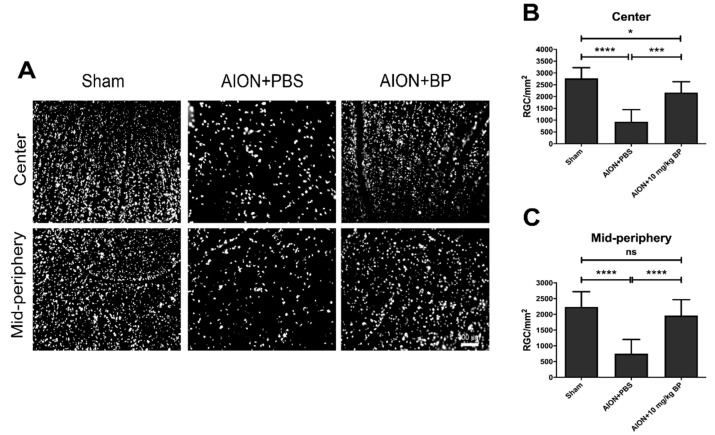
The RGC density in the whole retina was evaluated in the rAION model to detect the therapeutic effects of BP. Qualitative images of the central and mid-peripheral retina in each group (**A**,**B**). Quantitative graphs of RGC density in the central retina in the sham, PBS-treated, and BP-treated groups were 2771 ± 453/mm^2^, 935 ± 514/mm^2^, and 2172 ± 458/mm^2^, respectively (**C**), and those in the mid-peripheral retina were 2236 ± 485/mm^2^, 750 ± 452/mm^2^, and 1962 ± 505/mm^2^, respectively. (*, *p* ≤ 0.05; ***, *p* ≤ 0.001; ****, *p* < 0.0001; n.s., not significant; *n* = 6.)

To investigate the influence of BP on RGC survival rates after AION induction, retrograde labeling with fluorogold (FG) was used. The RGC counts of the sham group in the central and mid-peripheral retina were 2771 ± 453/mm^2^ and 2236 ± 485/mm^2^, respectively. After AION induction, the RGC counts of the PBS-treated and BP-treated groups in the central retina were 935 ± 514/mm^2^ and 2172 ± 458/mm^2^, respectively (Figure 1A,B). In addition, the RGC counts of the mid-peripheral retina in the PBS-treated and BP-treated groups were 750 ± 452/mm^2^ and 1962 ± 505/mm^2^, respectively (Figure 1A,C). In general, BP significantly improved the survival rates of the central and mid-peripheral retina in the rAION model, by 44.7% and 54.2%, respectively, suggesting that BP has neuroprotective effects on RGCs.

### 2.2. BP Preserved the Visual Function

**Figure 2:**
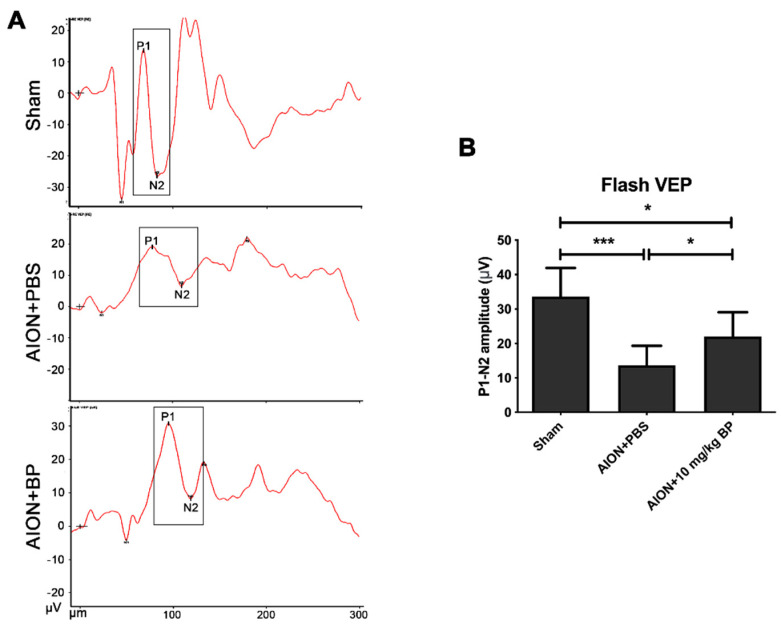
The amplitudes were recorded by FVEPs to evaluate the visual function. The images of electric potential in each group were shown in (**A**). Quantitative graphs of P1-N2 amplitudes in the sham, PBS-treated, and BP-treated groups were 33.63 ± 8.30 μV, 13.70 ± 5.59 μV, and 22.01 ± 7.03 μV, respectively (**B**) (***, *p* ≤ 0.001; *, *p* ≤ 0.05; *n* = 6).

To measure the voltage of P1-N2, the rats received visual light stimulation from flash visually evoked potentials (FVEPs). The amplitudes of the sham, PBS-treated, and BP-treated groups were 33.63 ± 8.30 μV, 13.70 ± 5.59 μV, and 22.01 ± 7.03 μV, respectively (Figure 2). These results demonstrated that BP can prevent the loss of visual function after ischemic injury.

### 2.3. BP Mitigated Optic Disc Edema and Preserved RNFL Thickness

**Figure 3:**
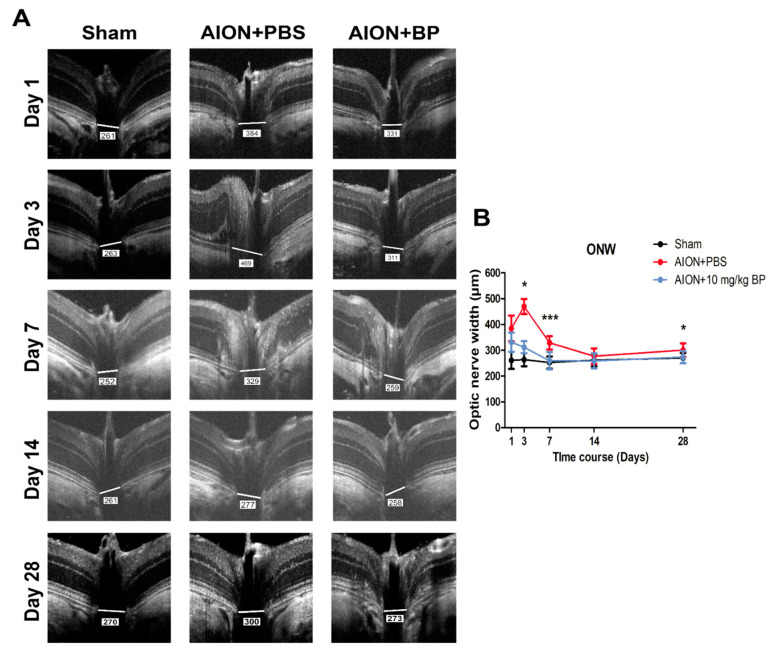
Imaging of optic nerve width by OCT scanning. OCT images of ONW in each group on days 1, 3, 7, 14, and 28 (**A**). The BP-treated group exhibited significantly alleviated optic disc edema on days 3, 7, and 28 compared with the PBS-treated group. (311.95 ± 23.21 μm versus 469.58 ± 29.35 μm, 259.35 ± 33.73 μm versus 329 ± 25.73 μm, and 273.71 ± 23.22 μm versus 300.73 ± 26.41 μm, respectively) (**B**) (***, *p* ≤ 0.001; *, *p* ≤ 0.05; *n* = 6).

**Figure 4:**
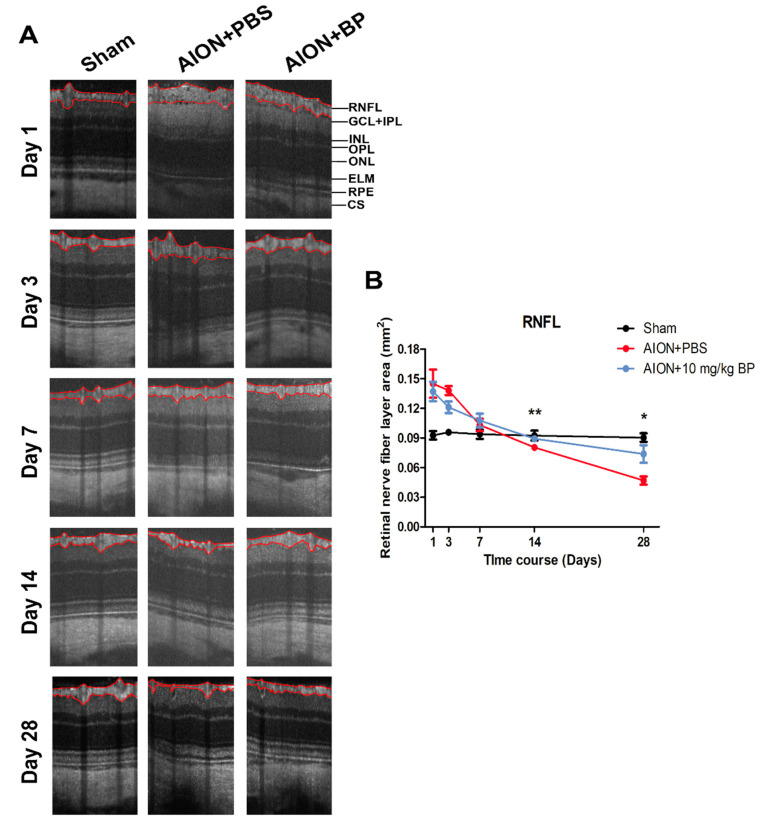
Image of the retinal nerve fiber layer by OCT scanning. OCT images of RNFL thickness in each group on days 1, 3, 7, 14, and 28 (**A**). The BP-treated group exhibited significant maintenance of RNFL thickness at days 14 and 28 compared with the PBS-treated group (0.089 ± 0.0027 mm^2^ versus 0.08 ± 0.0028 mm^2^ and 0.074 ± 0.0089 mm^2^ versus 0.047 ± 0.0042 mm^2^, respectively) (**B**) (**, *p* ≤ 0.01; *, *p* ≤ 0.05; *n* = 6. RNFL: retinal nerve fiber layer; GCL + IPL: ganglion cell layer + inner plexiform layer; INL: inner nuclear layer; OPL: outer plexiform layer; ONL: outer nuclear layer; ELM: external limiting membrane; RPE: retinal pigment epithelium; CS: choroidal stroma).

Optical coherence tomography (OCT) imaging was used to measure the optic nerve width (ONW) (Figure 3) and the retinal nerve fiber layer (RNFL) thickness (Figure 4) in the sham, PBS-treated, and BP-treated groups on days 1, 3, 7, 14, and 28. After AION induction, optic disc swelling was clearly observed in the acute stage and was significantly improved by BP administration on days 3, 7, and 28 (311.95 ± 23.21 μm versus 469.58 ± 29.35 μm, 259.35 ± 33.73 μm versus 329 ± 25.73 μm, and 273.71 ± 23.22 μm versus 300.73 ± 26.41 μm, respectively). The RNFL thickness profiles were measured, and the results showed that BP effectively preserved RNFL thickness compared with the PBS-treated group on days 14 and 28 (0.089 ± 0.0027 mm^2^ versus 0.08 ± 0.0028 mm^2^ and 0.074 ± 0.0089 mm^2^ versus 0.047 ± 0.0042 mm^2^, respectively).

### 2.4. BP Reduced Apoptotic Cells in the RGC Layer

**Figure 5:**
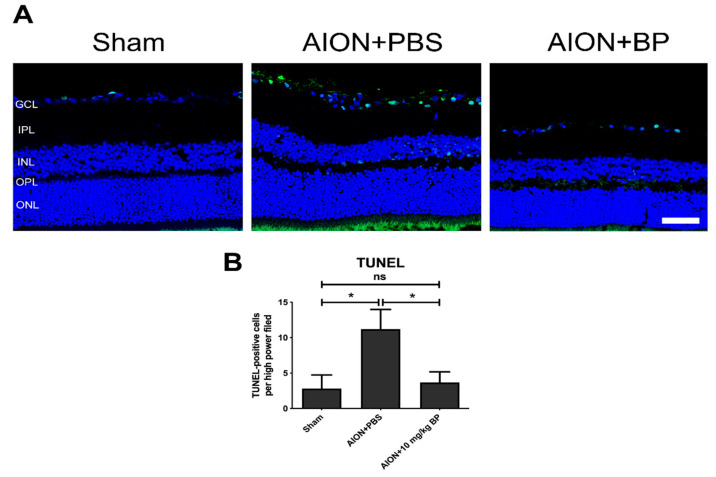
Detection of RGC apoptosis by TUNEL assay. TUNEL-stained image of retinal cross-sections in each group (**A**). Apoptotic cells were counted in the sham, PBS-treated, and BP-treated groups and were 2.8 ± 1.9, 11.2 ± 2.8, and 3.7 ± 1.5 TUNEL-positive cells/HPF, respectively. Scale bar = 50 μm (**B**) (*, *p ≤* 0.05; n.s., not significant; *n* = 6. GCL: ganglion cell layer; IPL: inner plexiform layer; INL: inner nuclear layer; OPL: outer plexiform layer; ONL: outer nuclear layer).

The apoptotic situation of the RGC layer was analyzed using a terminal deoxynucleotidyl transferase dUTP nick end labeling (TUNEL) assay. The quantities of TUNEL-positive cells/high powered field (HPF) in the sham, PBS-treated, and BP-treated groups were 2.8 ± 1.9, 11.2 ± 2.8, and 3.7 ± 1.5 TUNEL-positive cells/HPF, respectively (Figure 5). After AION induction, RGC apoptosis was significantly increased in the PBS-treated groups. However, the number of TUNEL-positive cells was decreased in the BP treatment group, suggesting that BP had an antiapoptotic effect in the rAION model.

### 2.5. BP Reduced Macrophage Infiltration into the Optic Nerve from the Blood

**Figure 6:**
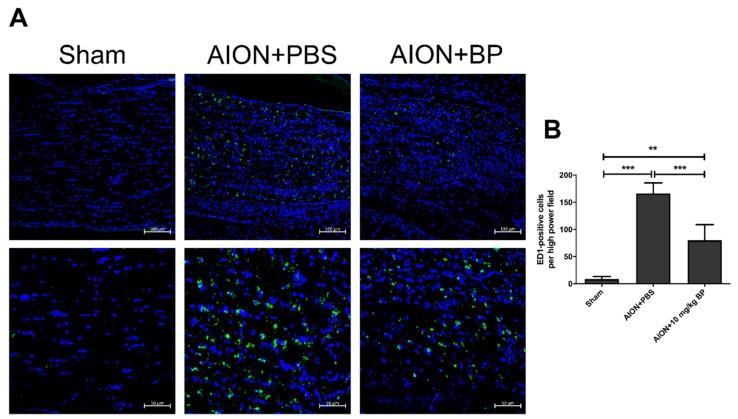
Immunostaining in the ON with ED1 antibody. ED1 immunostaining image of ON tissue in each group. The upper rows represent 10× magnification and the lower rows represent high (20×) magnification in the same specimen. (**A**). Quantitative analysis of ED1-positive cells (green) in the sham, PBS-treated, and BP-treated groups revealed 8.7 ± 4.8, 166.4 ± 19.3, and 80.1 ± 28.9 cells/HPF, respectively (**B**) (***, *p* ≤ 0.001; **, *p* ≤ 0.01; *n* = 6. The upper column of scale bar = 100 μm, the lower column of scale bar = 50 μm).

The inflammatory response of ED1^+^ blood-borne macrophages in ON after AION induction may cause ON damage [28]. Hence, we investigated whether BP inhibits ED1^+^ macrophage infiltration in the rAION model. The quantities of ED1-positive cells in the sham, PBS-treated, and BP-treated groups were 8.7 ± 4.8, 166.4 ± 19.3, and 80.1 ± 28.9 cells/HPF, respectively (Figure 6). The immunohistochemistry (IHC) results indicate that BP treatment had an anti-inflammatory effect by reducing ED1^+^ macrophage accumulation in ONs.

### 2.6. BP Preserved the Completeness of the Myelin Sheath in the rAION Model

**Figure 7:**
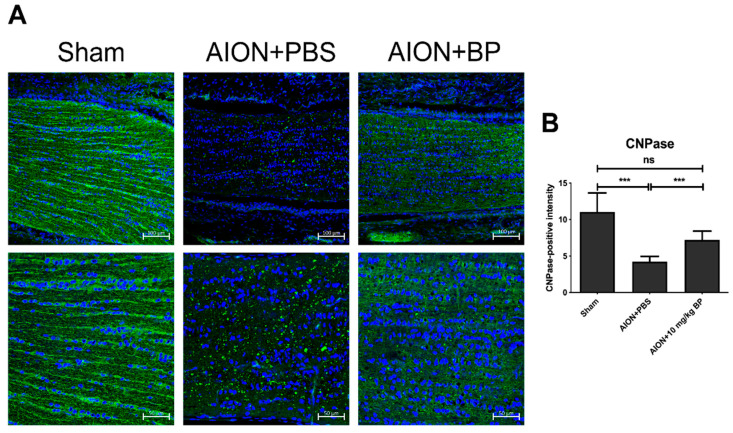
Expression of CNPase in optic nerve. Optic nerve sections reveal the intensity of CNPase in each group. The upper rows represent 10× magnification and the lower rows represent high (20×) magnification in the same specimen. (**A**). The results of quantitative analysis of CNPase+ intensity (green)/DAPI in the sham, PBS-treated, and BP-treated groups were 11.06 ± 2.60, 4.24 ± 0.71, and 7.20 ± 1.22, respectively (**B**) (***, *p* ≤ 0.001; n.s., not significant; *n* = 6. The upper column of scale bar = 100 μm, the lower column of scale bar = 50 μm).

2′,3′-Cyclic nucleotide 3′-phosphohydrolase (CNPase) participates in the process of myelination and is an important component of the cytoskeleton in oligodendrocyte cells [29,30]. Demyelination is one of the primary characteristics of many neurodegenerative diseases [31,32,33,34]; therefore, we investigated the levels of CNPase in the rAION model to evaluate the completeness of the myelin sheath. IHC results demonstrated that CNPase signaling was highly expressed in the sham group (11.06 ± 2.60). After AION induction, significantly lower expression of CNPase (4.24 ± 0.71) and reduced damage to the myelin structure in ON tissue were observed. In contrast, BP treatment simultaneously increased the expression of CNPase (7.20 ± 1.22) and preserved the integrity of the myelin sheath (Figure 7). In brief, the process of demyelination and myelin dysfunction was prevented by BP treatment in the rAION model.

### 2.7. BP Inhibited the Inflammatory Response through the NF-κB Signaling Pathway

**Figure 8:**
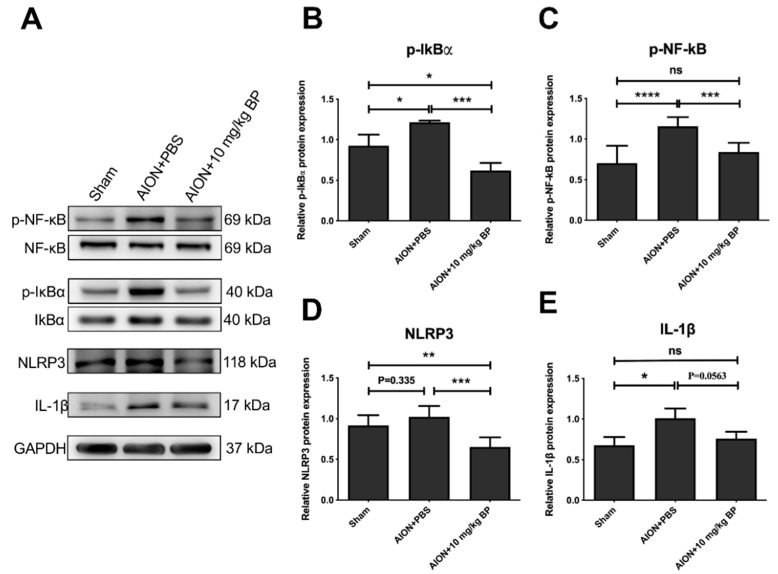
Western blotting revealed the molecular mechanisms of BP treatment in the rAION model. Qualitative blot images of NF-κB, p-NF-κB, IκBα, p-IκBα, NLRP3, IL-1β, and GAPDH (**A**). Bar graphics display the relative density of each protein signal (**B**–**E**). Values of the sham group were set to 1 (****, *p* ≤ 0.0001; ***, *p* ≤ 0.001; **, *p* ≤ 0.01; *, *p* ≤ 0.05. n.s., not significant).

Previous studies have shown that the upregulation of reactive oxygen species (ROS) in ischemic injury induces an inflammatory response through the IκBα-NF-κB signaling pathway [35]. Accordingly, we evaluated the protein expression of the NF-κB-associated pathway to investigate the molecular mechanisms of the neuroprotective effect of BP treatment. In the rAION model, high expression of phospho-IκBα induces phospho-NF-κB translocation to the nucleus, which can activate inflammatory gene transcription and NLRP3 and IL-1β signaling. However, compared with AION+PBS group, the BP-treated group exhibited significantly inhibited phosphorylation of IκBα and NF-κB (Figure 8A–C) and reduced expression of downstream inflammatory cytokines, NLRP3 and IL-1β (Figure 8A,D,E). Taken together, these results demonstrate that BP is capable of neuroprotective effects through modulation of the NF-κB inflammatory signaling pathway in the rAION model.

## 3. Discussion

In this study, we observed that BP can promote the survival rates of RGCs after AION induction based on the morphologic results of fluorogold labeling. Furthermore, the electric potential results of FVEP showed a favorable phenomenon of preserving visual function by using BP treatment in cases of ischemic injury. According to the immunostaining results, we determined that BP can effectively prevent ED1-positive macrophage infiltration into ON tissue, thereby mitigating the response to neuroinflammation and improving the outcome of demyelination.

Oxidative stress plays a critical role in the pathogenesis of several eye diseases, including neurodegenerative processes and neuroinflammatory responses, and is one of the main causes of irreversible injury [36,37,38]. In age-related retinal diseases, it has been found that the imbalance between the production of ROS and the antioxidant defense response can cause cell damage and ultimately lead to loss of vision [39,40]. Previous studies have indicated that high levels of ROS can induce the NF-κB signaling pathway when ischemic and hypoxia injury occur [35,41,42], leading to downstream proinflammatory factors activating an increased response of inflammatory cells [43,44]. Thus, the regulation of oxidative stress-associated pathways is crucial for the development of ischemic injury and cell survival. In this study, we found that BP can inhibit the activation of IκBα and NF-κB in the rAION model, suggesting that BP may potentially modulate the outcomes of oxidative stress.

Although we have confirmed that BP can effectively prevent extrinsic macrophage infiltration, the neuroinflammation caused by intrinsic microglia cannot be ignored in the central nervous system (CNS). In persistent or exaggerated neuroinflammation, microglia damage neurons through the excess production of cytotoxic factors, as demonstrated in many neurodegenerative diseases [45,46,47,48]. Microglial activation and recruitment after AION induction may result in optic nerve damage [28,49]. Notably, NF-κB signaling-mediated activation of the NLRP3 inflammasome is a key determinant of the development of neuroinflammation in microglia [50,51]. Our immunoblotting results showed that treatment with BP markedly inhibited the protein expression of NLRP3; therefore, we believe that BP can mitigate the neuroinflammation of microglia by downregulating the NF-κB-mediated NLRP3 inflammasome.

In optic neuritis, the loss of axons caused by the process of demyelination leads to severe dysfunction, which is closely related to changes in the visual field and the thickness of the RNFL [52,53]. Several studies in an experimental autoimmune disease model reported that inflammatory cells released a neurotoxic cytokine attack against the myelin sheath, resulting in further loss of RGCs in apoptosis [54,55]. However, our IHC staining results showed that BP can prevent the process of demyelination by inhibiting inflammatory cell activation, and RGC apoptosis was also significantly reduced, as demonstrated by the results from the TUNEL assay.

Steroids have been used in the preclinical study of AION [56] and are currently a common clinical treatment, which can significantly improve acute inflammation to preserve patients’ vision [2,57]. However, the administration of steroids was also associated with an increased risk of various ocular conditions, including glaucoma, cataracts, and crystalline keratopathy [58,59,60]. In this study, we confirmed that n-Butylidenephthalide was effective in reducing neuroinflammation after AION induction, and no adverse reactions were observed in rats. Nevertheless, the clinical results still need to be further examined. Although we explored the neuroinflammation of AION, the complete mechanism that constitutes the cause of this chain reaction is still unknown. Thus, we did not exclude other effects on different targets in AION. The detailed pathogenic mechanism of AION remains to be investigated.

According to the results of this research and those of previous reports [43,50,51,61], the regulation of NF-κB-mediated inflammation is crucial for neuroinflammation and demyelination after AION induction. In addition, maintaining the integrity of the myelin sheath by inhibiting the development of inflammation can significantly improve the survival rates of RGCs and preserve visual function. Thus, we think that NF-κB inhibitor administration to NAION patients is a potential treatment strategy in the future.

## 4. Materials and Methods

### 4.1. Animals

All the animal experiments were approved by the Institutional Animal Care and Use Committee (IACUC) at Tzu Chi University. Animal experimental procedures in vision research were implemented on the basis of the ARVO statement.

**Figure 9:**
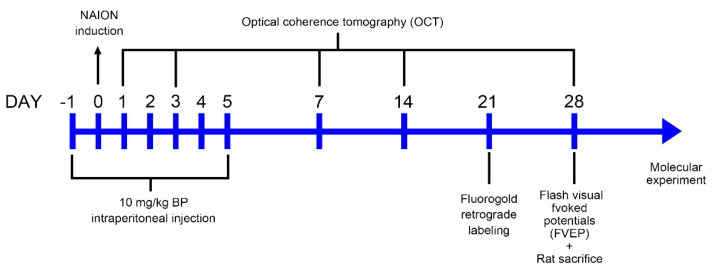
Timeline of the experimental process.

Four- to six-week-old adult male Wistar rats (100–125 g weight) (BioLASCO Taiwan Co., Ltd., Taiwan) were used for the ischemic optic neuropathy model. The three groups in this study were as follows: sham, treatment with PBS after AION injury, and AION injury treated with 10 mg/kg BP (purity ≥ 95%, Alfa Aesar, Thermo Fisher Scientific, Waltham, MA, USA) by intraperitoneal injection on seven consecutive days. The rats were bred in an animal room with a 12 h light–dark cycle (7 AM–7 PM light cycle time), controlled at a temperature of 23 °C and 55% humidity. We furnished the rates with filtered sterilization water and normal rodent chow. The general anesthesia of the rats during the experimental process was conducted using ketamine (100 mg per kg) and xylazine (10 mg per kg) injected intramuscularly. Local anesthesia was conducted using 0.5% Alcaine (Alcon, Puurs, Belgium), and pupil dilation was induced by administering Mydrin-P (Santen Pharmaceutical Co., Ltd., Osaka, Japan). The sacrifice process of rats was conducted using CO_2_ with a filling rate at 5 L/min, which is 20% of cage volume, with the primary goal of minimizing animal suffering. The timeline of the experimental process is illustrated in Figure 9.

### 4.2. AION Induction

First, the Alcaine and Mydrin-P drops were administered to the rats under local anesthesia to induce pupil dilation. An intravenous injection of rose bengal (2.5 mM in pH 7.4 PBS) (1 mL per kg) (Sigma-Aldrich, St. Louis, MO, USA) was then administered, and then immediately excited throughout the optic disc by exposure to an argon green laser (532 nm wavelength, 500 mm size, and 80 mW power) at 12 pulses (1/s). The laser was concentrated on the optic disc using a fundus lens. After this, Tobradex eye ointment (Alcon, Fort Worth, TX, USA) was spread evenly on the eyes of the rats. The physical health of the rats was cared for daily until the research had been finalized.

### 4.3. Retrograde Labeling of RGCs with Fluoro-Gold (FG)

Retrograde labeling was performed 3 weeks after the rats were AION induced. The sagittal region of the skull was taken as the positioning coordinates, and 2 μL of 5% Fluorogold was administered to the superior colliculus (AP: −6 mm; ML: ±1.5 mm; DV: −4 mm) by injection. The rats were sacrificed 1 week after labeling, and the eyeballs were exteriorized carefully and fixed in 10% formalin for 2 hrs. The whole retina was flat-mounted on a slide for morphological examination via fluorescence microscope (Axio Scope A1, Carl Zeiss AG, Oberkochen, Germany) using a filter set (excitation filter: 350~400 nm; emission filter: 515 nm). Distances of 1 mm and 3 mm from the center-radius of the retina were defined as a central and mid-peripheral area, respectively. Evaluation and counting of the RGC density was conducted based on at least 10 randomly selected regions (38,250 μm^2^; 225 μm × 170 μm) in the central and mid-peripheral areas to determine the RGC survival rate.

### 4.4. Flash Visual Evoked Potential (FVEP)

At day 28 after AION induction, with the sagittal region of the skull taken as the positioning coordinates, electrodes were implanted into the primary visual cortex region (AP: −8 mm; ML: ±3 mm) and frontal cortex (AP: +1 mm). The ground electrode was planted on the tail. The visual electrodiagnostic system (Espion, Diagnosys LLC, Gaithersburg, MA, USA) was set as follows: no background Illumination; a ganzfeld flash intensity of 0 db; a single flash rate of light of 1.9 Hz; an artifact rejection threshold of 20 mV; and a sampling rate of 2000 Hz. Measurements were taken as the average of 100 sweeps. The entire journey of the recording process was carried out in a dark room. The overview of visual functions was evaluated via P1 to N2 amplitude.

### 4.5. Immunohistochemistry (IHC)

First, the ON vertical-sections were blocked using 2% bovine serum albumin (BSA) containing 0.3% triton X-100 for 1 h. ON tissue was incubated with anti-ED1 (1:50; Bio-Rad Laboratories, Inc., Berkeley, CA, USA) and anti-2′, 3′-cyclic nucleotide 3′-phosphodiesterase (CNPase) (1:100; Abcam, Cambridge, UK) primary antibodies overnight at 4 °C. Goat anti-mouse Alexa Fluor 488 antibody (1:100, Invitrogen, Waltham, MA, USA) was used as a secondary antibody and incubated with the section for 1 hour at room temperature (RT). Fluorescent images of the ON tissue section were taken at 10x and 20× magnification using a Zeiss LSM 900 confocal system (Carl Zeiss AG, Oberkochen, Germany). The 20× magnification image of ED1^+^ cell was analyzed using ImageJ software for quantization of extrinsic macrophages.

### 4.6. Terminal Deoxynucleotidyl Transferase dUTP Nick End Labeling (TUNEL) Assay

Apoptotic cells in the ganglion cell layer (GCL) were detected using TUNEL assay in accordance with manufacturer’s protocol (DeadEndTM Fluorometric TUNEL System; Promega Corporation, Madison, WI, USA). TUNEL-positive cells were manually counted, based on at least six retina sections of each eyeball in three groups (*n* = 6 for each group).

### 4.7. Optical Coherence Tomography (OCT) Imaging

Coherence tomography images of optic nerve width (ONW) and retinal nerve fiber layer (RNFL) were taken using a Micron IV retinal microscope (Phoenix Technology Group, Campbell, CA, USA) on day 1, 3, 7, 14, and 28 post-AION induction. The imaging system was set to a longitudinal and transverse resolution of 1.8 μm and 3 μm, respectively, which provides a view of the retina of 3.2 mm and a 1.2 mm depth of field. The corneas were moistened with Methocel 2% (OmniVision GmbH, Puchheim, Germany). The Micron eyepiece was positioned in direct contact with the eye using gel. In order for the light to vertically penetrate the cornea, the RNFL and Bruch’s membrane opening (ONW) were imaged with circular and linear scans, respectively, which an average of 50 frames per scan.

### 4.8. Western Blotting Analysis

The detailed process of Western blotting was described in our previous reports [8,50]. The total protein was extracted from rat retina using a modified radioimmunoprecipitation (RIPA) buffer, and detection of protein concentration was conducted using a bicinchoninic acid (BCA) protein assay kit. The 50 μg retinal protein extracts were separated on 8% or 10% sodium dodecyl sulphate-polyacrylamide gels (SDS-PAGE), then transferred to polyvinylidene difluoride (PVDF) membranes. The membranes were blocked using a buffer containing 5% non-fat milk in TBST (0.02 M Tris-base, pH 7.6, 0.8% NaCl, 0.1% Tween 20) for 2 h at RT and then incubated overnight with anti-NF-κB (1:500; Abcam), anti-Phospho-NF-κB (1:200; Abcam), anti-IκBα (1:1000; Cell Signaling Technology, Inc., Danvers, MA, USA), anti-Phospho-IκBα (1:500; Cell Signaling Technology, Inc., Danvers, MA, USA), anti-NLRP3 (1:200; Novus Biologicals, Centennial, CO, USA), anti-IL-1β(1:200; Abcam), and anti-GAPDH (1:5000; Sigma-Aldrich, St. Louis, MO, USA) primary antibodies at 4 °C. The membranes were washed using TBST, and then incubated with corresponding horseradish peroxidase (HRP)-conjugated secondary antibody (1:10,000, Bio-Rad, Hercules, CA, USA) at room temperature for 2 h. The protein signaling on the membrane was detected using enhanced chemiluminescence (ECL) kits (RPN2232, GE Healthcare, Piscataway, NJ, USA). The signaling intensity of bands was quantitated using ImageJ software.

### 4.9. Statistical Analysis

The experimental results are presented as mean values ± standard deviation (SD). The Kruskal–Wallis test was carried out using the statistical comparisons software GraphPad Prism (GraphPad Software, Inc., La Jolla, CA, USA). The level of statistical significance was defined as *p* values < 0.05.

## 5. Conclusions

**Figure 10:**
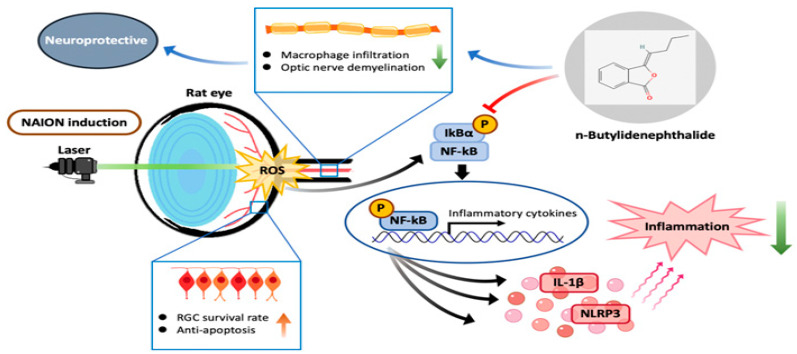
Summary of the neuroprotective effects of BP after AION induction.

In summary, this study indicates that BP prevents retinal ganglion cells from undergoing apoptosis and preserves visual function by reducing macrophage infiltration, preventing the process of demyelination, and inhibiting inflammatory cytokine activation. Our results demonstrate the neuroprotective effects of BP through its modulation of the NF-κB pathway (Figure 10).

## Data Availability

All data generated or analyzed during this study are included in this article.
